# Metal Uptake by Birches and Scots Pines Grown on a Porcelain Landfill

**DOI:** 10.3390/molecules30102196

**Published:** 2025-05-17

**Authors:** Michaela Zeiner, Viktor Sjöberg, Helena Olsman

**Affiliations:** 1 Man-Technology-Environment Research Centre, School of Science and Technology, Örebro University, Fakultetsgatan 1, 70182 Orebro, Sweden; 2 Eurofins European Competence Centre for PFAS, Eurofins Food & Feed Testing Sweden AB, Sjöhagsgatan 3, 53140 Lidkoping, Sweden

**Keywords:** bioaccumulation, Lidköping (Sweden), metal uptake, porcelain brownfield, Scots pine, silver birch

## Abstract

Potentially toxic elements (PTEs) have steadily become a serious environmental problem, especially regarding brownfields chosen for reuse, e.g., as a residential area. “Norra Hamnstaden” in Lidköping (Sweden) has a long history of industrial activity, including porcelain production with the resultant industrial waste deposited close by resulting in elevated levels of metals used for porcelain glazes, especially lead. To estimate the bioavailability of twelve PTEs (As, Ba, Pb, Cd, Co, Cu, Cr, Mn, Mo, Ni, V, Zn), their uptake by birches (*Betula pendula*) as well as Scots pines (*Pinus sylvestris*) was investigated through analyzing their leaves. Sampling was carried out on five trees once per month in the period from May to August. Different uptake patterns were observed for birches and pines, for the latter even varying with age. The birch samples showed higher contents of nickel, cobalt, molybdenum, and lead compared to the reference trees. Also, the pine needles had elevated lead levels, although by a lower factor. Birch leaves revealed surprising patterns of elevated element bioaccumulation factors, with barium reaching up to eight, offering the possibility to limit analyses to plant material for risk assessments instead of soil analysis.

## 1. Introduction

The western harbor in Lidköping, the so-called Hamnstaden, is an industrial area nowadays also with a sewage treatment plant and a small marina. In the 20th century, its northern part was used as landfill for the waste from the surrounding industries, such as a porcelain factory [1]. Due to plans for reusing this brownfield for residential areas, risk assessments including soil and ground water analysis alongside bioavailability studies are needed and ongoing. The soil-polluting elements arsenic, cadmium, chromium, copper, lead, nickel, and zinc are of particular concern for residents via different uptake routes, such as soil inhalation, dermal contact, and direct/indirect ingestion [2].

The porcelain industry in Norra Hamnstaden started in 1892 with the painting of imported porcelain [3]. In 1912, the production of feldspar-based porcelain started [3] which is pre-fired at 900 °C, glazed, and finally fired at 1400 °C. The starting materials mainly consist of alumina and iron silicates, but also kaolinite, illite, and chlorite [4]. Furthermore, the clay composition contains various impurities, such as quartz, calcium and magnesium carbonates, alkali and alkaline earth metal oxides, and different iron compounds, which differ a lot even when taken from the same site [4]. Nevertheless, the ratio of certain trace elements can be used for locating production sites of archeological porcelain findings [5]. Another contribution to the elemental composition of such products is the glazing of the porcelain surface by applying a thin outer glass layer, its main constituent being silica (SiO_2_). To lower the melting point and to improve the glaze’s properties, different metal oxides are added, e.g., white lead (2PbCO_3_*Pb(OH)_2_) [4,6]. Various metal salts are added to the glaze for coloring, mainly containing the following eight metals, titanium, vanadium, chromium, manganese, iron, cobalt, nickel, and copper, but in rare cases also compounds of gold, uranium, cadmium, antimony, and selenium are used [4]. Leaching of coloring salts from properly prepared glazes is not of concern [7], but porcelain waste stored in the environment is supposed to undergo degradation processes which might lead to leaching of potentially toxic elements to the surroundings [8]. Furthermore, it has been found that aging of contaminated soil increases the bioavailability of certain elements, e.g., lead [9,10]. Consequently, the number of the potentially polluting elements as well as their contents are expected to vary widely within the deposit and need to be investigated as the basis for site-specific risk assessment. The present investigation focused on the bioavailability of selected elements via monitoring of tree leaves. Besides foliar uptake, metals, nonmetals, as well as metalloids enter plants via roots, thus the elemental pattern from the soil is reflected in these plant parts [11,12,13]. Whilst essential elements need to be taken up for plant physiology and to avoid deficiencies [14,15], this uptake might be interfered by non-essential, even potentially toxic elements using similar uptake and storage pathways. During the vegetation period, harmful elements are accumulating in leaves so that they can be removed in autumn [16]. Metal uptake from soil into plants depends on various factors, one being the metal load in the surroundings, i.e., the soil composition and the pollution of the respective site [17,18,19,20]. Furthermore, the characteristic climatic conditions of each site which even changes from year to year [16] determine the obtained elemental contents in a plant tissue. Apart from these influencing parameters, analyzing various plants grown in the same area has shown that the uptake and accumulation of elements is species-specific [21,22]. In addition to these influencing parameters, the plants’ strategies to minimize either the uptake of potentially toxic elements or, later on, toxicity by these elements, are manifold [23,24,25], thus making the overall interpretation more complex.

**Figure 1:**
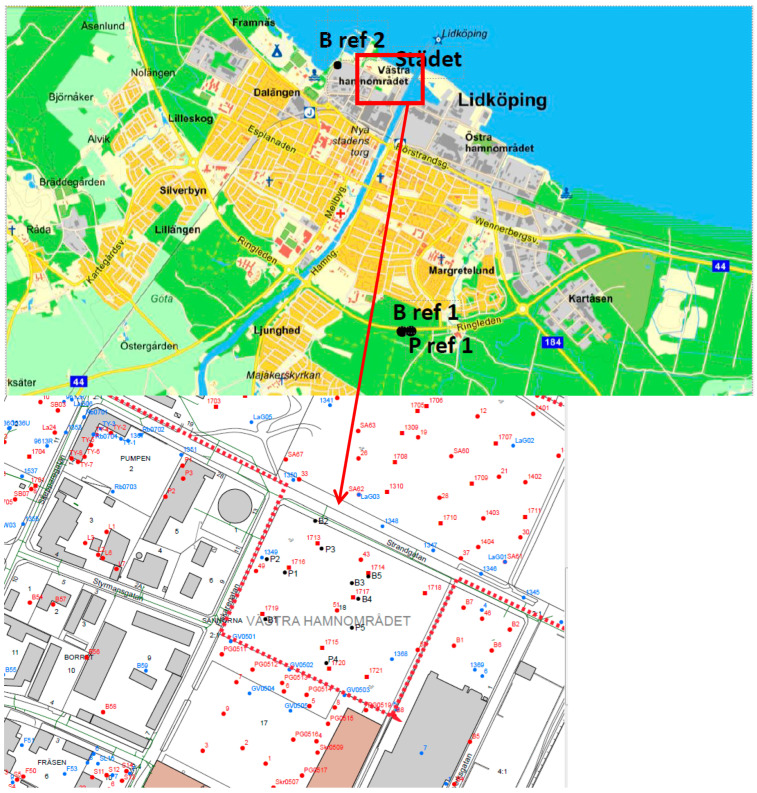
Study area and tree sampling plan for reference trees (BR1, BR2, PR1) and contaminated area (birches B1–B5, pines P1–P5) [graphics provided by Jordnära Miljökonsult].

The area of interest has already been studied, but these previous investigations carried out between 1999 and 2013 were mainly focused on total soil contents of certain contaminants [1], but for local risk assessment studies the actual bioavailability, e.g., via plant uptake, is needed. Thus, for the present investigation, two species were chosen, namely a deciduous tree birch (*Betula pendula*) as well as a conifer Scots pine (*Pinus sylvestris*) which both grow on the area of interest, i.e., the old porcelain deposit (see Figure 1). Birches are medium-sized deciduous trees found in Europe, parts of Asia, but also in America. As a pioneer species it grows after a forest fire or on bare land among the first tree species [26]. Scots pines are the most widely distributed pine tree species and used for monitoring environmental pollution. Like birches, Scots pines are pioneer species that can colonize nutrient-poor soils, e.g., contaminated sites [26,27]. Another advantage of this tree is that needles can reach a higher age than birch leaves, thus the accumulation can be studied over a longer period [22,28].

## 2. Results and Discussion

### 2.1. Figures of Merit of the Analytical Procedure

The applicability of the chosen method for the given analysis was proven by the results obtained for the analyzed standard reference material. Accuracy expressed by trueness (as recovery in %) and precision (as RSD in %) for all analytes obtained by analyzing the pine needle standard reference material NIST 1575a is given in Table 1. Overall, the recoveries for the elements with certified as well as with reference mass fractions were in the range of 86% up to 105%, their relative standard deviations being all below 3%. All external calibration curves had R^2^ values beyond 0.997.

### 2.2. Elemental Contents in Birch Leaves

The results obtained for the birch leaves from the contaminated site along with those for the two reference trees are shown in the following charts. For each tree, the determined elemental contents are given as mean values in mg/kg dried leaf. All mass fractions obtained for the trees grown in the contaminated area are compared with those for the reference trees using a *t*-test based on 95% probability level; all calculated *p*-values are shown in Table 2. Thus, all values <0.05 stand for statistically significant differences. Whilst Pb and Co differ for all trees from the brownfield, none up to three differ for the other elements.

**Figure 2:**
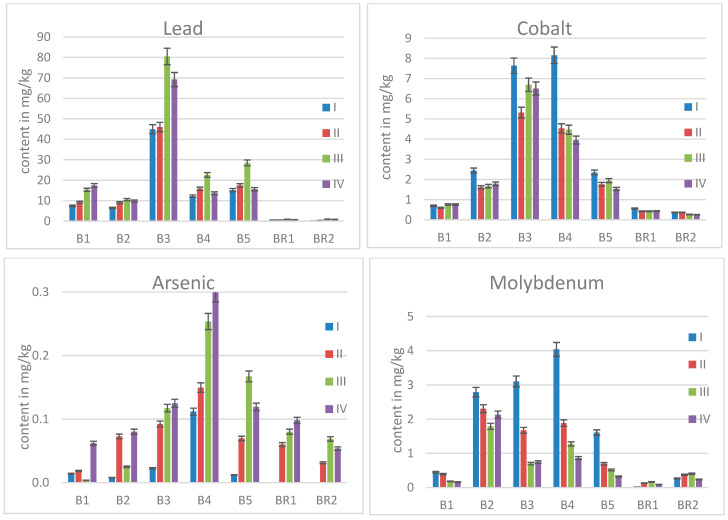
Mean contents of Pb, Co, As, and Mo in birch leaves for all sampling times.

Lead was detected in soil samples from the area of interest in contents up to 52 × 10^3^ mg/kg [29]. Birches are not considered as hyper-accumulators regarding Pb, i.e., accumulating more than 1 × 10^3^ mg/kg of this element in the leaves [30]. Nevertheless, statistically significantly elevated mass fractions were found in all five trees from the contaminated site (see Figure 2). The Pb levels rise during the vegetation period since trees tend to get rid of toxic elements in fall by loss of the leaves. Even if all trees from the contaminated site accumulated more Pb than the reference trees, no direct correlation to the soil content of this metal or the age/stem diameter of the trees could be found. Birches B1 and B3 had similar stem diameters. Sample B1, however, collected close to the soil sample with the highest Pb content, did not exhibit the highest content in the leaves, even containing less than B3 located in a less contaminated part of the site (Pb up to 7 × 10^3^ mg/kg). Furthermore, tree B4, located close to B3 and with greater stem diameter, took up less Pb. This site-specific uptake can be explained by the fact that the metal uptake is not limited to one soil sampling spot, but the roots of trees are widespread and grow more towards less contaminated areas to avoid high uptake of harmful elements [31].

Also, for cobalt, higher uptake can be stated for trees grown on the contaminated soil (see Figure 2). However, in contrast to Pb, Co is essential for plants, being required by various enzymes [32]. The soil Co contents of the sampling site were, in decreasing order, 380 mg/kg (for B2) > 89 mg/kg (for B5) > 60 mg/kg (for B1) > 43 mg/kg (for B3 and B4) [1,29]. Looking at the order of the Co contents in the respective leaves, this order is not reflected, which is determined to be B3, B4 > B2, B5 > B1.

For arsenic, the soil contents did not vary a lot, but all data from the respective sampling spots were in the range from 6 mg/kg to 10 mg/kg. Nevertheless, the contents in the birch leaves were not that evenly distributed; B4 in particular had accumulated more As than the other trees (see Figure 2). Leaching tests on porcelain waste showed that no harmful effects are expected to the surrounding soil, surface water, and groundwater environment [33]; however, elevated soil As contents are stated for the study area [29] and also reflected in the leaves.

The findings for molybdenum are also shown in Figure 2. Regarding this element, an accumulation can be stated for all trees grown in the contaminated area, except for B1, whose Mo level was in the range of that found for the reference trees. The trend of decreasing Mo content with time was registered for all trees B1 to B5 in contrast to no significant change during the vegetation period in the leaves of the reference trees. This accumulation behavior is typical for essential micronutrients, which Mo is [34], but particularly obvious when more of the respective essential element is present.

**Figure 3:**
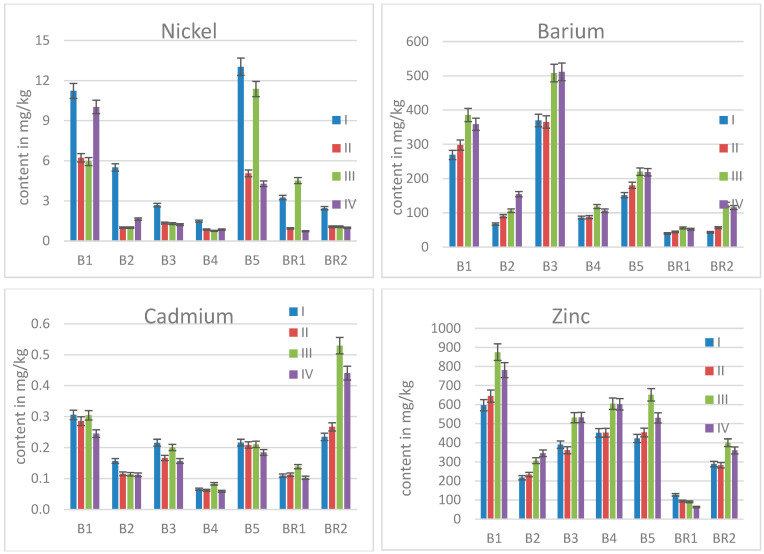
Mean contents of Ni, Ba, Cd, and Zn in birch leaves for all sampling times

A diverse picture was obtained for nickel (Figure 3). Whereas the soil Ni contents were all found to be in a comparable range, namely from 19 mg/kg to 28 mg/kg [1,29], the contents in the leaves varied widely. B1 and B5 contained statistically significant more Ni than B2, B3, and B4 whose Ni levels were in the range of the reference trees.

Elevated contents for barium (Figure 3) were found in all sampled trees from the contaminated area, whereby the leaf contents for B2 and B4 were close to those of the reference trees. In all cases, an increase in time was found as expected for a non-essential element. Ba is the only element with an accumulation factor (content in leave/content in soil) beyond 1 for all trees B1 to B5 (Table 3). This ratio is sometimes also called soil-to-plant transfer factor [35].

Cadmium, a toxic metal to plants, was found in higher amounts in the leaves of the reference trees than in those from the contaminated site (compare Figure 3), the order of the contents being B4 < BR1, B2 < B3, B5 < B1 < BR2. Looking at the accumulation factors, values up to approx. 1 are determined for Cd (Table 3).

The results for Zn show that this metal was present in significantly lower amounts in the leaves of BR1 than in B1 to B5, but the values determined for BR2 were in the same order of magnitude as B2 to B5 (Figure 3). Only B1, showing the highest contents at all sampling times, differs from both reference trees significantly. Accumulation of elements strongly depends on the surroundings and characteristics of the site. Sitko and colleagues found site-specific elevations for Mn or Zn in birch leaves at different post-industrial heaps in Poland [36]. Dresler and colleagues investigated silver birch trees near Zn-Pb ore-processing sites, finding higher accumulation of trace metals in leaves, which further affects the accumulation of phenolic acids and flavonoids [37]. The dust in the smelter vicinity is rich in heavy metals, thus not only root uptake of the elements of interest is to be expected, as at the present study location of the porcelain brownfield.

In contrast to the above discussed elements, no accumulation trends in the leaves from B1 to B5 in comparison to BR1 and BR2 were found for copper, vanadium, and chromium.

Based on the contents in the collected birch leaves, the respective accumulation factors were calculated for As, Ba, Co, Cu, Cr, Ni, Pb, V, and Zn for the samples collected in July which are presented in Table 3. The soil contents for the elements in a depth of 0.5 m to 1 m of the nearest soil sampling spot(s) to each sampled tree were used. These factors can only provide an estimation of the metal uptake since the roots of the trees are further distributed down than the depth of 1 m and not only close to the soil sampling site, which is even explicitly shown by B3 having a different soil sampling spot in the close vicinity. To avoid a high uptake of harmful elements, plants produce more and longer roots to more favorable regions [31] alongside other strategies to combat (heavy) metal toxicity, such as restricting metals to cell walls, vascular sequestration, and synthesizing biochemical compounds to minimize toxic effects [23]. This wide range of possibilities in handling toxic elements by plants explains the variations in the calculated accumulation factors and clearly show that the soil composition is only one parameter in the whole uptake and storage mechanism. Kozlov and coworkers found in their study on uptake roots of heavy metals by birch in an industrially polluted area that nickel, but not copper, effectively translocates from roots to shoots and leaves in mountain birch, with most contamination occurring due to dust particles on leaf surfaces [24]. This is reflected in the accumulation factors obtained in the present study for those elements listed in Table 3. The plant–soil relationship of various metals was investigated by Pająk and coworkers stating that Scots pine needles and silver birch and leaves show increased accumulation of Zn and Pb in response to Pb–Zn ore mining and processing plants, but not Cu, Cd, and Cr [26].

### 2.3. Elemental Contents in Scots Pine Needles

The pine needles were analyzed for the same elements as the birch leaves, and even when growing on the same contaminated area, the uptake and accumulation behavior is quite different, underlining the species specificity of these processes as already stated in literature [19]. The calculated *p*-values for each element determined in the fresh needles are shown in Table 4, to allow comparability with the birch leaves which only last one vegetation period. Lead, even if found in higher amounts than in the reference needles, does not differ significantly in any of the trees after one summer. Like for the birch leaves, Mo is accumulated to a significant extent in almost all sampled pine needles.

In contrast to deciduous trees, conifers offer the possibility to investigate long-term metal accumulation, since the needles of certain species can reach an age up to seven years. This fact was used in the present study by sampling and analyzing needle samples of three different ages. Even if no statistically significant differences in the mass fractions for Pb were found for the fresh roots, the 1-a and 2-a-old needles accumulated enough of this metal to differ significantly from the needles from the reference trees. The *p*-values for the *t*-tests are 0.0040, 0.19, 0.013, 5.0 × 10^−7^, and 0.038 for the 1-a-old needles for P1, P2, P3, P4, and P5, respectively, and 0.00042, 0.099, 0.0042, 6.2 × 10^−8^, and 0.0018 for the 2-a-old needles.

**Figure 4:**
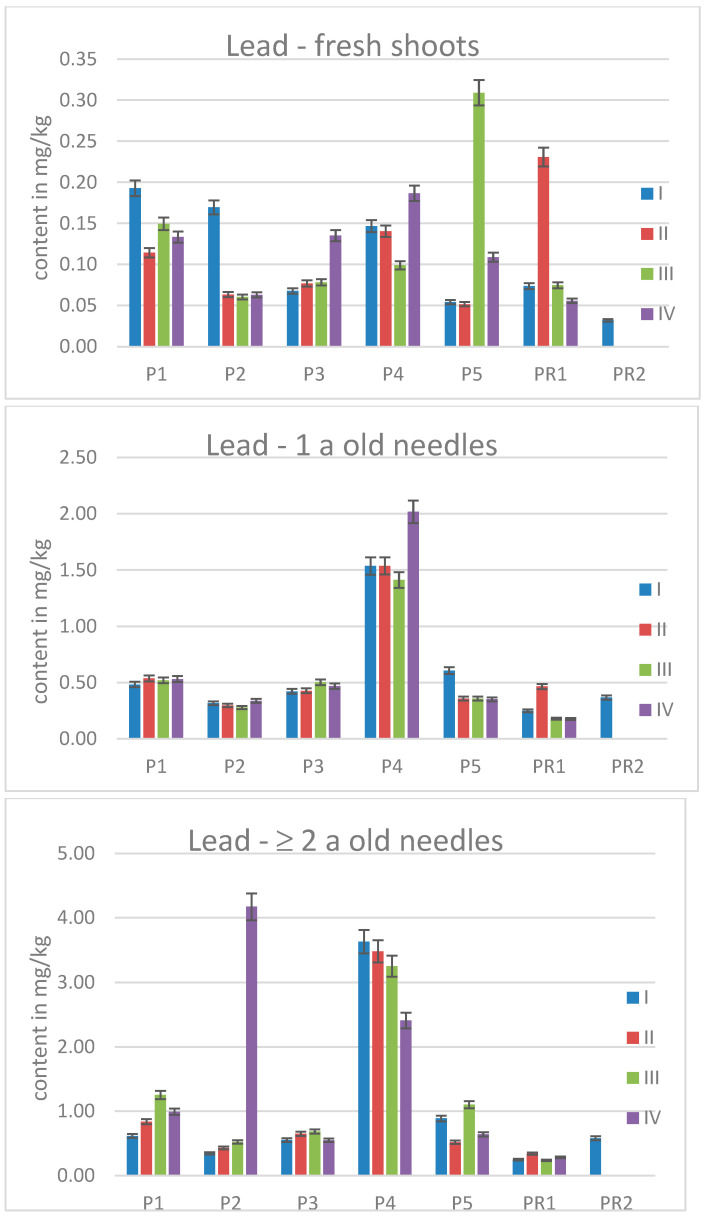
Lead content in pine needles of different ages for all sampling times.

As already seen for the birch leaves, lead, as one of the main pollutants of interest, was found in the trees growing at the contaminated site in higher contents than in the reference tree(s), even if the difference is not as obvious as for the birch leaves. This harmful metal is accumulated in the needles over time, as can be seen from the values increasing with needle age (compare Figure 4). The determined Pb mass fractions for P4 are close to the data of P1 for the fresh shoots, but much higher for the one-year-old and even older needles. P4 grows close to the soil sampling spot with the lowest Pb soil levels (20 mg/kg to 280 mg/kg; [29]) compared to the other pine sampling places (Pb ranging from 1.3 × 10^3^ mg/kg up to 52 × 10^3^ mg/kg; [1,29]). This finding of the highest Pb levels in P4 seems to be of interest regarding metal uptake from soil. Even if the needles were washed prior to sample preparation to avoid metal contamination from precipitation, there is always a certain percentage of pollutant taken up via the stomata as well as via cuticular cracks [11]. A study of Scots pine needles in a mining area found a strong plant–soil relationship for Pb [26]. Çomaklı and colleague published that Scots pine needles are the plant tissue with the highest accumulation factors for many metals [38].

**Figure 5:**
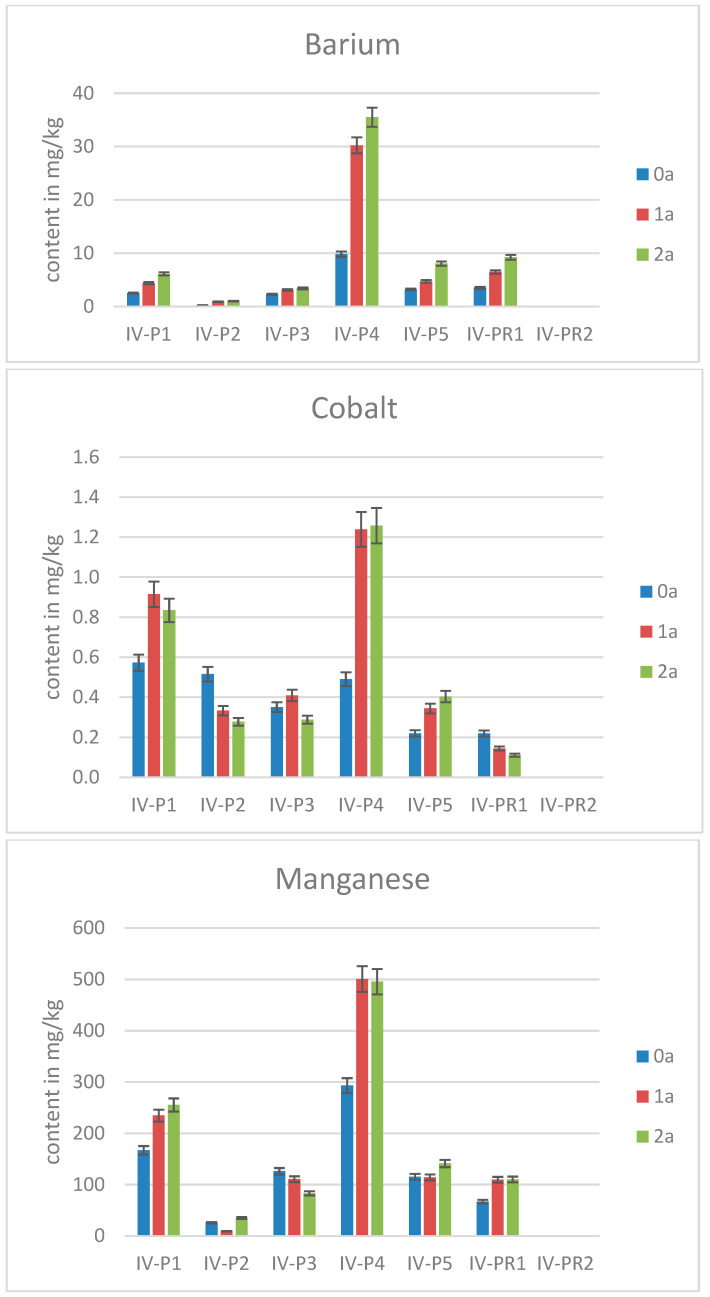
Barium, cobalt, and manganese in pine needles sampled in August.

Not only for Pb, but also for other metals, namely Co, Mn, and Ba, P4 shows the highest contents, followed by P1. For illustration, the results for these three analytes are given in Figure 5 for the sampling time August (IV). In contrast, Cd was found in statistically higher contents only in P3 and P4. Regarding historical municipal solid waste landfill soils in Bratislava, Slovakia, which show high metal (loid) concentrations, Cd was determined as being the most bioaccessible [39].

Whereas the results for As and Zn differed in the birch leaves; no significant differences between pines from the contaminated and reference sites were found. In general, the obtained data for the pine needles are in the same order of magnitude than those for various pine species grown in the Lisičine Arboretum in Croatia [22].

Soil composition is an important parameter influencing the uptake and accumulation of (toxic) metals, but plants become tolerant through various strategies, like a mineral toxicity fence. It has been found that magnesium and iron levels in Scots pine needles play a crucial role in regulating heavy metal fluxes, which contribute to their survival and response to pollution effects [40].

### 2.4. Comparison of Birches and Scots Pines

As already pointed out in the respective subchapters on birch leaves and Scots pine needles, both show different uptake and accumulation behavior of the elements studied. Not only the absolute mass fractions found, but also the comparison to the reference trees reflect this fact. Regarding Pb, up to 80 mg/kg was found for birches for B3, with the other trees showing leaf Pb mass fractions around 6 mg/kg to 30 mg/kg, whilst the needles contain much less after one, two, or even three and more vegetation periods, the maximum being around 4 mg/kg. Both birches and pines are used as bioindicators for metal contamination, but they differ in their mechanisms and efficiency of metal uptake and translocation. Birches tend to accumulate metals more from surface deposition and show distinct root-to-shoot translocation patterns, while pines primarily reflect soil metal concentrations and exhibit species-specific internal distribution. As shown by Kozlov et al., a significant portion (>80%) of nickel and copper in birch foliage comes from dust deposition on leaf surfaces, with only a minor part removed by washing, indicating strong canopy uptake [24]. For pine needles, surface deposition is less emphasized compared to birches [26]. In addition to the primary uptake root, internal translocation and distribution determines the mass fractions determined in the leaf tissues, the shoot-to-translocation being element-specific. Nickel is more effectively translocated from roots to shoots than copper, showing selective internal movement [24]. Internal transport of metals can be investigated using radionuclides like ^90^Sr and ^137^Cs; birches as well as pine show age-dependent internal distribution [41]. For detailed assessment of metal transfer and translocation in plants, so-called dynamic factors considering internal (physiological) as well as external (ecological) factors is recommended [42].

## 3. Materials and Methods

### 3.1. Sampling

Sampling was performed by a representative of Jordnära Miljökonsult once per month in the vegetation period from May to August 2018. In the following, these sampling times are indicated as I—May; II—June; III—July, and IV—August. As recommended by the ICP Forest Manual on sampling leaves and needles [43], five trees per species growing in the contaminated area were sampled (B1–B5, P1–P5) along with two reference trees (BR1, BR2, PR1, PR2). For *Pinus sylvestris*, the second reference tree was cut after the first sampling, thus later needles were collected only from one reference tree. The exact sampling locations are shown in Figure 1. From each birch tree, 15–20 leaves were collected each month. Each pine tree was sampled for fresh needles, one-year-old needles, and ≥two-year-old needles. Leaves and needles were pooled by sampling occasion, tree, and age prior to further treatment.

### 3.2. Sample Preparation and Elemental Analysis

To avoid falsification of the data by metals attached to the leaf or needle surface, all samples were rinsed three times with ultrapure water (18.2 MΩ) prior to the drying step at 105 °C. The dried plant material was then ground and homogenized. Three portions of each sample (approx. 100 mg, weighed to the nearest 0.1 mg) underwent acidic microwave-assisted digestion using nitric acid (65% *w*/*w* originating from Merck, Darmstadt, Germany) and hydrogen peroxide (30% *w*/*w* originating from Supelco, Darmstadt, Germany), with the following volumes used: for birch leaves 6 mL concentrated nitric acid, 3 mL deionized ultrapure water, and 1 mL hydrogen peroxide solution; for pine needles 6 mL concentrated nitric acid, 3 mL deionized ultrapure water, and 2 mL hydrogen peroxide solution. The instrument used was a CEM MarsV microwave oven. The temperature program included two steps with 20 min each (heating to 150 °C and 180 °C, respectively). The obtained clear solutions were filled up to 50 mL with ultrapure water (18.2 MΩ) from the own in-house water-purification facility. Prior to the measurement, each solution was filtered through a 0.2 µm polypropylene filter and the internal standard added (Rh with a final concentration of 10 µg/L). Blank solutions were prepared analogously.

For quality assurance (repeatability, trueness), six aliquots of the standard reference material SRM 1575a—Trace Elements in Pine Needles (Gaithersburg, MD, USA) were treated in the same way as the samples.

All obtained digest solutions including blanks were analyzed using inductively coupled plasma mass spectrometry (ICP-MS; Agilent 7500cx) after further dilution of 1:10 using 1% *w/w* nitric acid. The instrumental conditions applied are listed in Table 5. External calibration based on five multi-elemental standard solutions prepared from ICP multi-element standard solution VI (Merck, Darmstadt, Germany) was used for quantification.

### 3.3. Data Evaluation and Statistical Tests

All raw data from the instrument (mass concentration of the analytes) were converted into contents in mg/kg considering digestion blank, final volume, dilution step, and mass of sample digested. The mean and standard deviation was calculated for each sample based on the triplicate digestion. Microsoft Office Excel, version 2016 was used to perform the calculations as well as statistical tests. *t*-tests were applied to check for statistical significance of differences between elemental content determined in leaves from contaminated site vs. those from reference trees (based on *p* = 0.05 as limit). Accumulation factor calculations are based on the soil sampling site closest to the respective tree; in the case of B3, two spots were similar in distance, thus both were considered. The formula used was content of element in plant material divided by the total content of the element in the respective soil sample, whereby the plant content is always calculated on a dry matter basis.

## 4. Conclusions

Based on the results obtained for birch leaves and pine needles sampled in the contaminated site at the porcelain deposit in Lidköping (Sweden), elevated bioavailability compared to reference sites can be stated. Differences in the accumulation tendencies in leaves were found for As, Ba, Pb, Co, Cu, Cr, Ni, V, and Zn. Furthermore, different uptake patterns were observed for birch leaves and pine needles, whereby the latter also show a variation with increasing needle age. These differences highlight the importance of species selection and understanding uptake pathways when using trees as bioindicators of metal pollution. Comparison of the elemental contents in the plant tissues and the respective soil samples showed the limited significance of a direct correlation to soil data due to wide distribution of roots in the soil along with strategies of the plants to avoid uptake of harmful elements. The accumulation factors are mainly below 1; the highest values (up to 8) were obtained for Ba.

## Figures and Tables

**Table 1:** Recoveries and RSD for all analytes determined by SRM analysis.

Analyte	Recovery (%)	RSD (%)
As	101	2.9
Ba	86.2	2.3
Cd	98.9	1.4
Co	85.9	2.6
Cu	96.0	1.5
Cr	105	2.9
Mn	90.9	1.8
Mo	n/a *	n/a
Ni	96.1	2.5
Pb	91.2	2.8
V	n/a *	n/a
Zn	88.4	2.7

* not applicable, since not certified in the pine needle SRM.

**Table 2:** *p*-values for *t*-tests between each birch tree (B1–B5) and the reference trees (BR1 + BR2); statistically significant differences are marked bold (*p* < 0.05).

Analyte ↓/Tree →	B1	B2	B3	B4	B5
As	**0.030**	0.33	0.30	**0.0054**	0.38
Ba	**1.0 × 10^−6^**	0.10	**4.6 × 10^−7^**	0.097	**0.00011**
Cd	0.62	0.19	0.52	0.064	0.67
Co	**0.00025**	**7.9 × 10^−7^**	**3.5 × 10^−9^**	**2.0 × 10^−5^**	**2.9 × 10^−7^**
Cu	0.12	0.87	**0.0022**	**0.039**	0.85
Cr	0.29	0.61	0.23	0.61	0.32
Mo	0.34	**1.3 × 10^−7^**	**0.0056**	**0.0036**	**0.018**
Ni	**0.00021**	0.70	0.76	0.25	**0.0025**
Pb	**2.7 × 10^−5^**	**8.4 × 10^−8^**	**1.5 × 10^−6^**	**1.6 × 10^−6^**	**5.1 × 10^−6^**
V	**0.00058**	0.68	0.77	0.13	0.32
Zn	**8.7 × 10^−5^**	0.41	**0.0095**	**0.0018**	**0.0029**

**Table 3:** Accumulation factors for birch leaves (bold values are > 1) based on elemental contents in the leaves collected in July and one to two soil sampling sites close to the tree-growing area (soil sampling site 1719 for B1; 1713 for B2; 1717 for B3a and B4; 1714 for B3b and B5).

Analyte ↓/Tree→	B1	B2	B3a	B3b	B4	B5	Min	Max	Mean
As	0.0007	0.0027	0.0198	0.0206	0.0428	0.0293	0.0007	0.0428	0.0193
Ba	**2.9171**	**6.6390**	**7.8302**	**3.6299**	**1.8243**	**1.5727**	**1.5727**	**7.8302**	**4.0689**
Cd	0.6614	0.5701	0.2255	**1.0033**	0.0936	**1.0532**	0.0936	**1.0532**	0.6012
Co	0.0126	0.0044	0.1574	0.0752	0.1050	0.0219	0.0044	0.1574	0.0627
Cu	0.0325	0.1159	0.0419	0.0635	0.0492	0.1147	0.0325	0.1159	0.0696
Cr	0.0423	0.0453	0.0135	0.0223	0.0128	0.0094	0.0094	0.0453	0.0243
Mo	0.3924	n/a *	0.3763	n/a *	0.6851	n/a *	0.3763	0.6851	0.4846
Ni	0.2631	0.0527	0.0466	0.0684	0.0274	0.5987	0.0274	0.5987	0.1762
Pb	0.0008	0.0015	0.0160	0.0136	0.0045	0.0048	0.0008	0.0160	0.0069
V	0.0224	0.0222	0.0029	0.0076	0.0019	0.0134	0.0019	0.0224	0.0117
Zn	**1.2641**	0.7482	0.0772	0.6480	0.0879	0.7940	0.0772	**1.2641**	0.6032

n/a *: not applicable, since no soil Mo data available.

**Table 4:** *p*-values for *t*-tests between fresh needles of each pine tree (P1–P5) and the reference trees (PR1 + PR2); statistically significant differences are marked bold (*p* < 0.05).

Analyte ↓/Tree→	P1	P2	P3	P4	P5
As	0.12	0.99	0.35	0.83	0.50
Ba	0.87	0.52	0.56	**1.5 × 10^−5^**	0.51
Cd	0.13	0.86	**2.4 × 10^−5^**	**0.0022**	0.23
Co	**0.0012**	**0.026**	0.13	**0.0030**	0.13
Cu	0.96	0.27	0.44	0.66	0.30
Cr	0.094	0.95	0.76	0.67	0.14
Mo	**0.010**	**0.0016**	**0.00026**	**0.0030**	**0.0012**
Ni	0.60	0.38	0.37	**0.0024**	0.096
Pb	0.054	0.49	0.46	0.066	0.23
V	0.61	0.51	0.49	0.50	0.85
Zn	0.56	0.18	0.16	0.69	0.11

**Table 5:** Instrumental conditions for analytical method used.

Parameter	ICP-MS
Instrument	Agilent 7500cx ICP-MS (Agilent, Tokyo, Japan)
Output power	1500 W
Argon flows	Plasma:15 L min^−1^ Auxiliary: 0.9 L min^−1^ Nebulizer: 0.2 L min^−1^
Sample flow	0.3 mL min^−1^
Nebulizer	MicroMist
Spray chamber	Scott double pass
Isotopes	^51^V, ^53^Cr, ^55^Mn, ^59^Co, ^60^Ni, ^63^Cu, ^66^Zn, ^75^As, ^95^Mo,^111^Cd, ^137^Ba, ^204+206+207+208^Pb
Collison cell	Off: Ba, Pb, Cd, Co, Cu, Mn, Mo, Ni, Zn On (He 5 mL/min): V, Cr, As

## Data Availability

All data will be available on reasonable request.
